# Nandrolone decanoate–induced hypogonadism in male rats: Dose‐ and time‐dependent effects on pituitary and testicular hormonal functions

**DOI:** 10.14814/phy2.70053

**Published:** 2024-10-06

**Authors:** Sholeh Karimi, Negar Kazori, Sayyed Mohammad Hadi Alavi, Sara Alijanpour, Mohammad Alim Atif Siddiqi, Bahman Zeynali

**Affiliations:** ^1^ Department of Animal Biology, School of Biology, College of Science University of Tehran Tehran Iran; ^2^ Present address: Department of Biology, Faculty of Education Loger Higher Education Institute Loger Afghanistan

**Keywords:** anabolic‐androgenic steroids, GSI, luteinizing hormone, sex steroids, testosterone enanthate

## Abstract

Anabolic‐androgenic steroids (AASs) impairment of reproduction has been reported. We investigated dose‐ and time‐dependent effects of Nandrolone decanoate (ND) on reproductive system in comparison with Testosterone enanthate (TE). Male Wistar rats were administrated with 1, 3, and 9 mg/kg/weeks ND or 1 and 3 mg/kg/weeks TE for 8 weeks, and testicular phenotype and reproductive hormones were assessed at 4 and 8 weeks post‐treatments. AASs × treatment period interaction was significant for gonadosomatic index (GSI), testosterone (T), 17β‐estradiol (E_2_), and luteinizing hormone (LH). At 4 weeks post‐treatment, GSI was decreased in rats treated with 3 mg/kg/weeks ND and T was decreased in all ND‐treated groups, while no significant changes in LH levels were observed. At 8 weeks post‐treatment, GSI was decreased in rats treated with 1 and 3 mg/kg/weeks ND and with 3 mg/kg/weeks TE, T was decreased in all groups, and E_2_ and LH were increased and decreased, respectively, in rats treated with 9 mg/kg/weeks ND and with 3 mg/kg/weeks TE. The testes showed histopathological defects in both ND‐ and TE‐treated rats suggesting a delay in seminiferous cycle. This study shows AASs‐induced hypogonadism at low‐dose that coincided with inhibition of T biosynthesis and disruption of T feedback on pituitary.

## Graphical abstract

Impacts of nandrolone decanoate (ND) were studied on pituitary‐testis axis in male rats, and compared with testosterone enanthate (TE). Results show ND‐ or TE‐induced hypogonadism coincided with inhibition of T biosynthesis, and suggest disruption of T negative feedback on pituitary. The testes showed histopathological defects in both ND and TE treated rats suggesting a delay in seminiferous cycle.

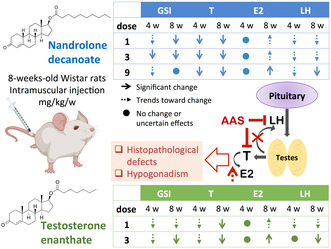

## 1 INTRODUCTION

Anabolic‐androgenic steroids (AASs) are synthetic derivatives of testosterone (T) with both anabolic and androgenic properties that have been synthesized in 1940s (Kicman, 2008; Kuhn, 2002). They were primarily used as pharmaceutical agents for clinical purposes to treat various illnesses such as anemia, sarcopenia, osteoporosis, and hypogonadism (Nieschlag & Nieschlag, 2012; Stojko et al., 2023). The therapeutic dose depends on age, type of illness, and type of AASs (Ganesan et al., 2019), which is 6–10 mg/days for hypogonadism on a continuous basis and with regular intervals of use (Hall & Hall, 2005). In case of nandrolone decanoate (ND), which is regularly used for treatment of anemia, the recommended therapeutic dose is 0.4 mg/kg/days for human (100–200 mg/weeks for 60 kg adult men) (Ganesan et al., 2019; Tamaki et al., 2003). About two decades later, abuse of AASs usually at high doses (10‐ to 100‐fold of therapeutic dose) spread among professional and non‐professional athletes for improving physical performance and muscle gain (Hartgens & Kuipers, 2004; Yesalis & Bahrke, 1995). In 1975, medical commission of the International Olympic Committee banned the use of AASs (Kanayama & Pope Jr., 2018; Pope Jr. et al., 2014). So far, frequent studies have shown impacts of AASs abuse on the male reproductive system (Table S1) indicating that their side effects depend on molecular structure, dose, and duration of administration. However, dose‐ and time‐dependent effects of AAS abuse at therapeutic level have been rarely studied.

AASs are membrane‐permeable that bind to androgen receptor (AR) to interfere with T signaling (Pope Jr. et al., 2014). Considering widespread distributions of AR in central and peripheral tissues, detrimental effects of AASs have been reported in many physiological systems, including reproduction (Albano et al., 2021; Gagliano‐Jucá & Basaria, 2019; Pope Jr. et al., 2014; Sessa et al., 2022). Using animal models, it has been shown that abuse of most AASs at ≥3 mg/kg/weeks decrease the mass of reproductive organs including testes or epididymis (Ahmed, 2015; Al‐Otaibi, 2024; Behairy et al., 2020; Breuer et al., 2001; Mesbah et al., 2007) along with decreases in sperm count and motility and increase in sperm with abnormal morphology (Table S1) (Abed et al., 2022; Al‐Otaibi, 2024; Alves et al., 2024; Behairy et al., 2020; Karbalay‐Doust et al., 2007; Mohamed & Mohamed, 2015; Shokri et al., 2014; Torres‐Calleja et al., 2000) (Table S1). The AASs‐induced hypogonadism and diminished sperm quality have shown to be associated with disruptions of hormonal functions of pituitary and testis (Table S1). In this context, it has been demonstrated that AASs cause apoptosis to the Leydig cells (Mesbah et al., 2008; Saddick, 2021) and decrease transcripts of genes encoding enzymes in the steroidogenesis pathway (Alsiö et al., 2009; Koeva et al., 2003; Min & Lee, 2018) resulting in decrease in circulating T level (Abed et al., 2022; Al‐Otaibi, 2024; Alves et al., 2024; Barone et al., 2017; Beutel et al., 2005; Ibrahim et al., 2022; Mohamed & Mohamed, 2015; Shahraki et al., 2015). It has also been reported that abuse of AASs increases circulating 17β‐estradiol (E_2_) level suggesting its estrogenic activity (Grönbladh et al., 2013; Selakovic et al., 2017). However, numbers of studies that investigated the effects of AASs abuse on the upstream regulators of steroidogenesis are rare, and current knowledge suggests decreases in gonadotropins levels either follicle stimulating hormone (FSH) or luteinizing hormone (LH) (Table S1).

The present study was designed to investigate dose‐ and time‐dependent adverse effects of ND abuse on reproductive system. We used ND because of its widespread abuse among professional and non‐professional athletes (Patanè et al., 2020; WADA, 2020). ND is metabolized to nandrolone (19‐nortestosterone), which exhibit higher binding affinity to AR compared to that of T (Brueggemeier, 2015; de Souza & Hallak, 2011; Kicman, 2008), particularly in tissues devoid of 5α‐reductase activity (Bergink et al., 1985). Vieira et al. (2008) reported a therapeutic dose of 0.7 mg/kg/weeks for rats, and determined 5.3 and 10.3 mg/kg/weeks as intermediate and suprapharmacological doses, respectively. Our literature survey also showed that ND‐induced hypogonadism, when it was abused at ≥3 mg/kg/weeks in rats via inhibition of T biosynthesis, resulted in diminished sperm quality (Table S1). However, the lower dose of ND has not been examined, and existing studies miss a positive control from AAS family to better elucidate ND effects on pituitary and testis hormonal functions.

In the present study, male Wistar rats were intramuscularly administrated with 1 (therapeutic dose), 3 (intermediate pharmacological dose), and 9 (supra‐pharmacological dos) mg/kg/weeks ND for 8 weeks, and testicular phenotypic changes and circulating levels of LH, T, and E_2_ were measured at 4 and 8 weeks post‐treatment. Groups of males were treated with testosterone enanthate (TE), which is metabolized to T for comparison (Brueggemeier, 2015; de Souza & Hallak, 2011). Our results show adverse effects of ND abuse on reproductive system at the therapeutic dose, and comparisons with TE provides novel information to better understand ND‐induced disruption of hormonal functions of the testes.

## 2 MATERIALS AND METHODS

### 2.1 Chemicals

Heparin, ND (CAS 360‐70‐3; Cat. No. Y0000548, Sigma) and TE (CAS 315‐37‐7; Cat. No. T3006, Sigma) were purchased from Alborz Darou Pharmaceutical Co. (Qazvin, Iran), Caspian Tamin Pharmaceutical Co. (Rasht, Iran), and Aburaihan Pharmaceutical Co. (Tehran, Iran), respectively. Benzyl alcohol (K47390359), formaldehyde solution 37% (44426802), diethyl ether (100930), paraffin (K49881157), xylene (K51663297), hematoxylin (C. I. 75290), and eosin Y (C. I. 45380) were purchased from Merck KGaA (Darmstadt, Germany). Ethanol was purchased from Kimia Alcohol (Zanjan, Iran).

### 2.2 Animals, AASs administration and sampling

**FIGURE 1:**
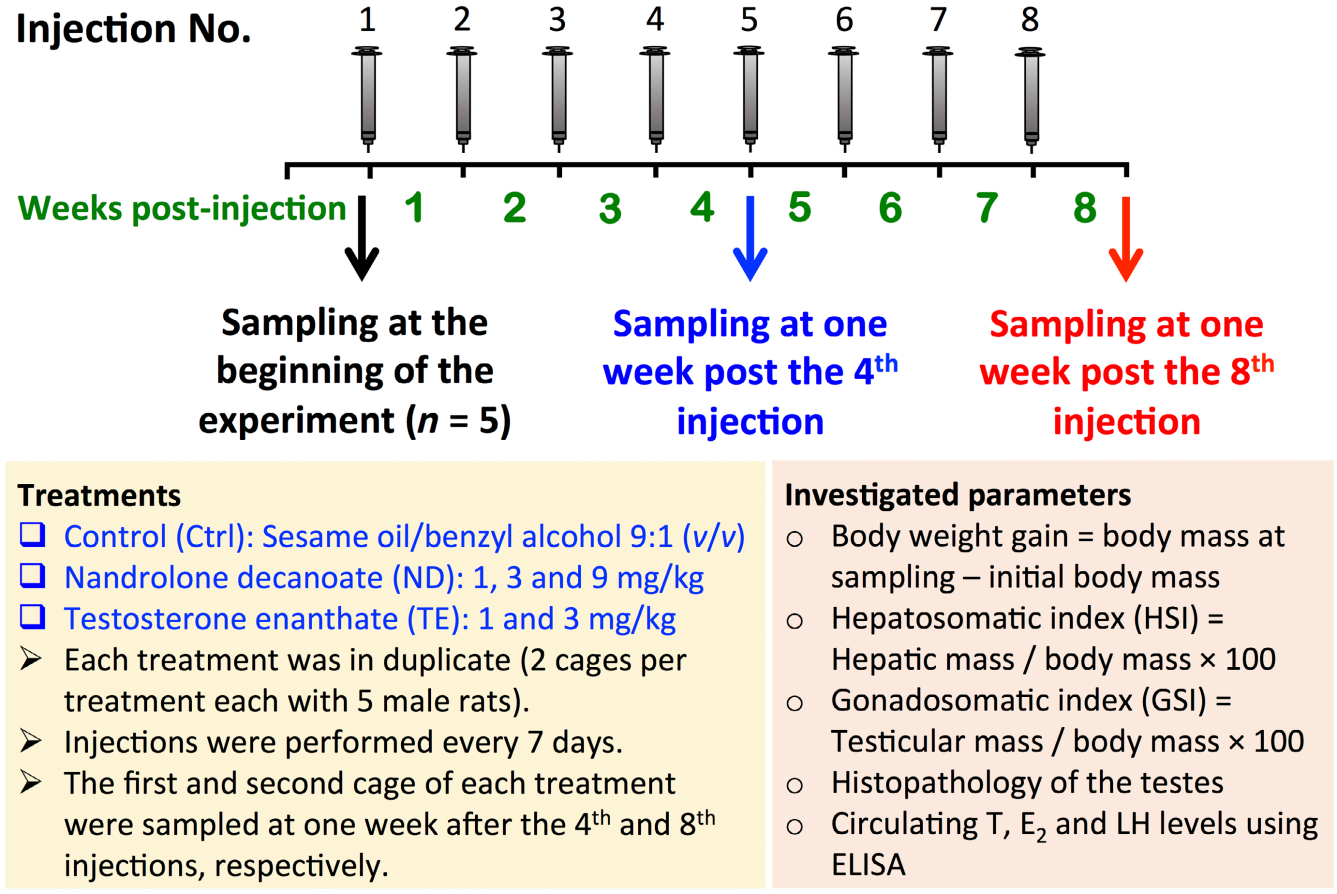
Experimental design of the present study. Adult male Wistar rats (8‐week‐old) were divided into six treatments including control (Ctrl), 1, 3, and 9 mg/kg/weeks nandrolone decanoate (ND), and 1 and 3 mg/kg/weeks testosterone enanthate (TE). For each treatment, five rats were sampled at 1 week after the fourth injection (cage # 1) and at 1 week after the eighth injection (cage # 2).

This study was performed at Animal Research Laboratory (ARL), Institute of Biochemistry and Biophysics (IBB), University of Tehran (UT), Tehran, Iran in 2020. Adult male Wistar rats (8 weeks old) were obtained from breeding colonies at ARL, IBB, UT, and divided into six treatments including control (Ctrl), 1, 3, and 9 mg/kg/weeks ND, and 1 and 3 mg/kg/weeks TE (Figure 1). Each treatment was composed of two replications each with five rats. One replicate from each treatment was sampled at 1 week after the 4th injection (4 weeks post‐treatment), and another replicate from the same treatment was sampled at 1 week after the eighth injection (8 weeks post‐treatment). The number of rats used in the present study was 60 (6 treatments × 2 replicates per treatment × *n* = 5 rats per replicate). The sample size was estimated 6.49 rats per treatment based on previous studies (Table S1) using pwr.anova.test, in which number of groups, significant level, power and effect size were 6, 0.05, 0.8, and 0.7, respectively. We “Reduced” number of animals to five rats per treatment due to very restriction rule of Ethics Committee at the UT. Interestingly, small variations in assessed parameters including gonadosomatic index (GSI), T and LH were observed among individuals in a same treatment (see results). There was an additional cage with five rats that were sampled 1 day before first AASs administrations to record reproductive characteristics at the beginning of the experiment.

Animals were maintained in a polycarbonate cage under controlled conditions with temperature of 20–22°C, humidity of 55%–65%, and light–dark cycle of 12:12 h. Animals were maintained together in a cage. The animals had free access to commercial food (Behparvar Co. Karaj, Iran) and water during the experimental period.

The ND (25 mg/mL) and TE (100 mg/mL) was diluted with sesame oil containing 10% benzyl alcohol (v/v) as solubilizer/preservative. ND and TE were administrated by intramuscular injection. ND and TE are long‐acting injectable forms of nandrolone and T with elimination half‐life of 6–12 (Minto et al., 1997) and 4.5 days, respectively (Behre & Nieschlag, 1998). While, free T is degraded with a half‐life of only 10 min (Zitzmann & Nieschlag, 2000). Considering the spermatogenic cycle in rats is estimated to be 54–56 days (Kolasa et al., 2004), we injected the rats with ND and TE once a week for 8 weeks according to previous studies (Table S1). Primary dilutions were conducted to inject the same volume of sesame oil (1 mL at each injection). The control group received the same volume of sesame oil containing 10% benzyl alcohol (v/v). One week after the fourth and eighth injection, the rats were sampled for body mass, tissues, and blood. The cages were cleaned twice per week, and rats were checked for their normal activity (such as mobility, drinking, and eating) and health (no skin inflammation, injury, and dirty body). One day before each injection, they were weighed to calculate the amount of AASs. Animals were tail marked and the weight gain (body mass at sampling—initial body mass) for each rat was assessed at 4 and 8 weeks post‐treatment.

Each sampling was performed in two successive days from 8:00 to 11:00 am. A rotation protocol was considered for sampling to minimize the circadian effects on hormonal levels. At first, one rat was sampled from all treatments, and then the second rat was sampled from all treatments until reaching the fifth rat. Diethyl ether was used to anesthetize the rats to minimize anesthesia effects on circulating reproductive hormone levels (Shomer et al., 2020). Anesthesia was checked by slow breathing rate and no response to pinch in the tail. When a rat anesthetized, the body mass was recorded, and immediately, the blood was collected from cardiac puncture using 20–22 gauge needles into a 5 mL syringe. The collected blood was poured into a 15 mL heparinized falcon to prevent clotting. The blood was centrifuged at 3000 rpm for 20 min at 4°C, and plasma was transferred into 1.5 mL Eppendorf tubes kept at −20°C until further analysis. Then, the animal was euthanized by cerebral dislocation followed by decapitation by guillotine. The testes and liver were weighed to assess (GSI = testicular mass/body mass × 100) and hepatosomatic index (HSI = liver mass/body mass × 100). The left testis was fixed for observation of histological defects. All efforts were made to reduce the number of animals, and to prevent their suffering from pain and distress.

### 2.3 Histology and seminiferous cycle

For light microscopy, testicular fragments were fixed in the 10% formalin for 72 h. Tissues were dehydrated in 70%, 80%, 90%, and 99.6% ethanol, cleared in xylene, and embedded in paraffin. Serial sections of 7 μm thickness were prepared using microtome (Erma, Tokyo, Japan) and stained with hematoxylin and eosin (H & E). The histological slides observed under the Nikon Labophot‐2 light microscopy using 10×, 25×, or 40× objective lenses. Histological images were captured using a HDMI+USB camera (KaiLiwei, China) mounted on the microscope, and were analyzed using ImageJ (https://imagej.net/ij/).

The stage of the seminiferous cycle was identified according to Leblond and Clermont (1952) and Morales et al. (2014). In the present study, Stages I–VI, Stages VII–VIII, and Stages IX–XIV were identified and pooled as single groups in the analyses. At Stages I–VI, spermatogonia, spermatocytes, early spermatids (called round spermatids), older spermatids characterized by elongated heads and dorsal fins, and immature spermatozoa are present. At Stages VII–VIII, the heads of the immature spermatozoa are fully developed and their flagella form whirls in the center. Spermatozoa may be released at Stage VIII. Spermatogonia are present and divided to produce a new generation of spermatocytes. The nuclei of spermatocytes are larger than Stages I–VI. At Stages IX–XIV, the spermatozoa have left and the nuclei of the spermatids begin flattening (Stage IX). During Stages X–XIV, the spermatids elongate and divisions of primary and secondary spermatocytes occur. The stage of the seminiferous cycle was determined for at least 20 seminiferous tubules per rat (*n* = 100 per treatment). Diameters of the tubule were also measured in round shape‐seminiferous tubules (*n* = 10 and 50 seminiferous tubules per rat and treatment, respectively), and the mean for each rat was used for statistical analyses.

### 2.4 Hormonal assessment

The rat LH ELISA kit (Cat. No. PTC‐10179‐R9648, ZellBio GmbH, Lonsee, Germany) was used to assay circulating LH level in the blood plasma according to manufacturer instructions. Forty microliter standard or samples and then 10 μL anti‐LH antibody labeled with biotin were added into each well of a 96‐well plate pre‐coated with anti‐LH monoclonal antibody. After adding 50 μL streptavidin‐HRP, the plate was incubated for 60 min at 37°C. The plate was then washed five times, 100 μL chromogen solution was added, and the plate was incubated in the dark for 10 min at 37°C. The reaction was stopped by adding 50 μL stop solution, and absorbance was read using at 450 nm. The sensitivity of the kit was 0.15 mIU/mL, and its assay ranged from 1.25 to 40 mIU/mL. The intra‐assay and inter‐assay coefficients of variance (CV) were <10% and 12%, respectively.

To measure gonadal steroids, T (Cat. No. 3725‐300A) and E_2_ (Cat. No. 4925‐300A) ELISA kits (Monobind Inc. Lake Forest, CA, USA) were used. For E_2_ assay, 25 μL of plasma samples and E_2_ standard were dispensed into each well of a 96‐well plate pre‐coated with anti‐E_2_ antibody. Anti‐E_2_ antibody (50 μL) labeled with biotin was added into each well and incubated for 30 min at room temperature (RT). Another 90 min incubation at RT was performed after adding 50 μL E_2_ enzyme reagent into each well. The plate was then washed three times, 100 μL substrate solution was added, and the plate was incubated in the dark for 20 min at RT. For T assay, 10 μL of plasma samples and T standard were dispensed into each well of a 96‐well plate pre‐coated with anti‐T antibody. T enzyme reagent (50 μL) and then 50 μL anti‐E_2_ antibody labeled with biotin were added into each well, and incubated for 60 min at RT. The plate was then washed three times, 100 μL substrate solution was added, and the plate was incubated for 15 min at RT. The reaction for T and E_2_ was stopped by adding 50 μL stop solution, and absorbance was read at 450 nm. Sensitivity of the E_2_ and T kits were 8.2 and 0.576 pg/mL, respectively. The intra‐assay and inter‐assay coefficients of variance (CV) were <9.9% and 8.2% for E_2_ and were <9.8% and 9.7% for T. Gonadal steroids were measured in duplicate for each sample and mean values were used in statistical analyses.

### 2.5 Data analysis

Data were tested for normality (Shapiro–Wilk test) and homogeneity of variance (Levene's test). Data were Log_10_ transformed to meet assumptions of normality and homogeneity of variance for circulating T level and T/E_2_ ratio. Two‐way analysis of variance (ANOVA) was used to understand the effects of AASs, treatment period and their interaction (Seltman, 2018). For parameters that had significant interaction (GSI and circulating T, E_2_, and LH levels), the models were revised into one‐way ANOVA models followed by Tukey's multiple comparisons test to investigate AASs effects on these parameters at each sampling time post‐treatment. When AASs × treatment period interaction was not significant (body weight gain, HSI, diameter of seminiferous tubules and T/E_2_ ratio), the models were revised into (a) one‐way ANOVA followed by Tukey's multiple comparisons test to investigate the main effects of AASs on these parameters, and (b) an independent *t*‐test to investigate the main effects of treatment period on these parameters. This study was not a repeated measure design, but composed of two groups that were sampled at 4 and 8 weeks post‐treatment. All statistical analyses were performed with GraphPad Prism 9.0.0 (GraphPad Software, San Diego, CA, USA). *p*‐value was set at 0.05 to indicate significant differences. All data are presented as mean ± standard deviation (SD).

## 3 RESULTS

There was no mortality and animals had normal activities during the experiment. Average (±SD) of body mass (g), HSI (%), GSI (%), T (ng/mL), E_2_ (ng/mL), and LH (mIU/mL) of rats at the time of first AASs administrations were 212.60 ± 33.94, 4.33 ± 1.29, 1.20 ± 0.35, 2.21 ± 1.62, 0.05 ± 0.04, and 0.89 ± 0.13, respectively.

### 3.1 Body mass, weight gain and HSI

**FIGURE 2:**
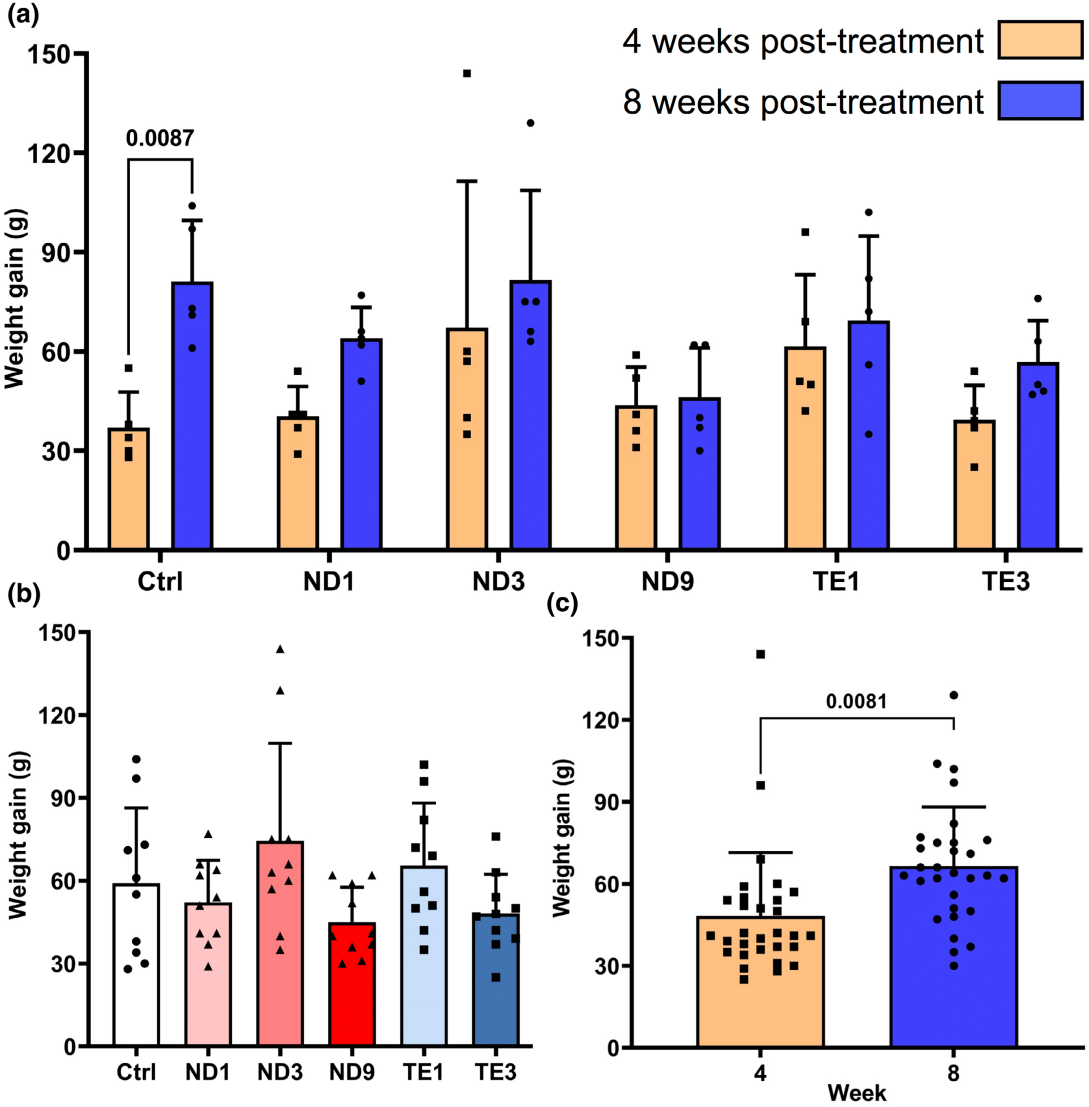
Nandrolone decanoate (ND) and testosterone enanthate (TE) abuses do not affect body weight gain in the male Wistar rats. Animals were weekly administrated with intramuscular injections of 1, 3, and 9 mg/kg ND and 1 and 3 mg/kg TE for 4 and 8 weeks. Body weight gain was calculated from the body mass at the beginning of the experiment and 1 week after the fourth injection (4 weeks post‐treatment) and eighth injection (8 weeks post‐treatment). Two‐way ANOVA showed non‐significant effects of AASs × treatment period interaction (a), thus the model was revised to study the effects of AASs (b) and treatment period (c) on body weight gain using one‐way ANOVA followed by Tukey's multiple comparisons test and independent *t*‐test, respectively. Data are shown as mean ± SD (*n* = 5). The actual *p* values are expressed.

In all groups, body mass was increased during the period of the experiment (Table S2). Two‐way ANOVA showed no significant AASs × treatment period interaction for body weight gain and HSI (Table 1; Figures 2a and S1A). Therefore, the models were revised to investigate the effects of main factors on these parameters. Body weight gain and HSI showed no significant differences among treatments (Figures 2b and S1B). However, body weight gain and HSI at 8 weeks post‐treatment were respectively higher (*p* = 0.0081) and lower (*p* = 0.0081 and *p* = 0.0023) than 4 weeks post‐treatment (Figures 2c and S1C).

**TABLE 1:** Summary of statistics (F_DFn, DFd_; *p*‐value) obtained from two‐way ANOVA models used to investigate the effects of anabolic‐androgenic steroids (AASs), treatment period, and their interaction on reproductive system in male Wistar rats.

	AASs	Treatment period	AASs × treatment period
Body weight gain	3.10_5, 20_; *p* = 0.1595	23.90_1, 4_; *p* = 0.0081	1.04_5, 20_; *p* = 0.4221
HSI	3.25_5, 20_; *p* = 0.7517	47.33_1, 4_; *p* = 0.0023	1.18_5, 20_; *p* = 0.3536
GSI	17.63_5, 20_; *p* < 0.0001	22.44_1, 4_; *p* = 0.0091	3.04_5, 20_; *p* = 0.0337
Diameter of ST	4.82_5, 20_; *p* = 0.2013	36.67_1, 4_; *p* = 0.1475	0.74_5, 20_; *p* = 0.6047
*Log* T	12.13_5, 20_; *p* = 0.0127	7.98_1, 4_; *p* = 0.0476	2.54_5, 20_; *p* = 0.0517
E_2_	1.06_5, 20_; *p* = 0.4140	0.72_1, 4_; *p* = 0.4431	2.76_5, 20_; *p* = 0.0473
*Log* T/E_2_	7.24_5, 20_; *p* = 0.0177	4.73_1, 4_; *p* = 0.0953	0.70_5, 20_; *p* = 0.5216
LH	12.18_5, 20_; *p* = 0.0020	17.19_1, 4_; *p* = 0.0143	4.41_5, 20_; *p* = 0.0535

Abbreviations: 17β‐estradiol, E_2_; gonadosomatic index, GSI; hepatosomatic index, HSI; testosterone, T; luteinizing hormone, LH; seminiferous tubules, ST.

### 3.2 Histopathology of the testes and GSI

**FIGURE 3:**
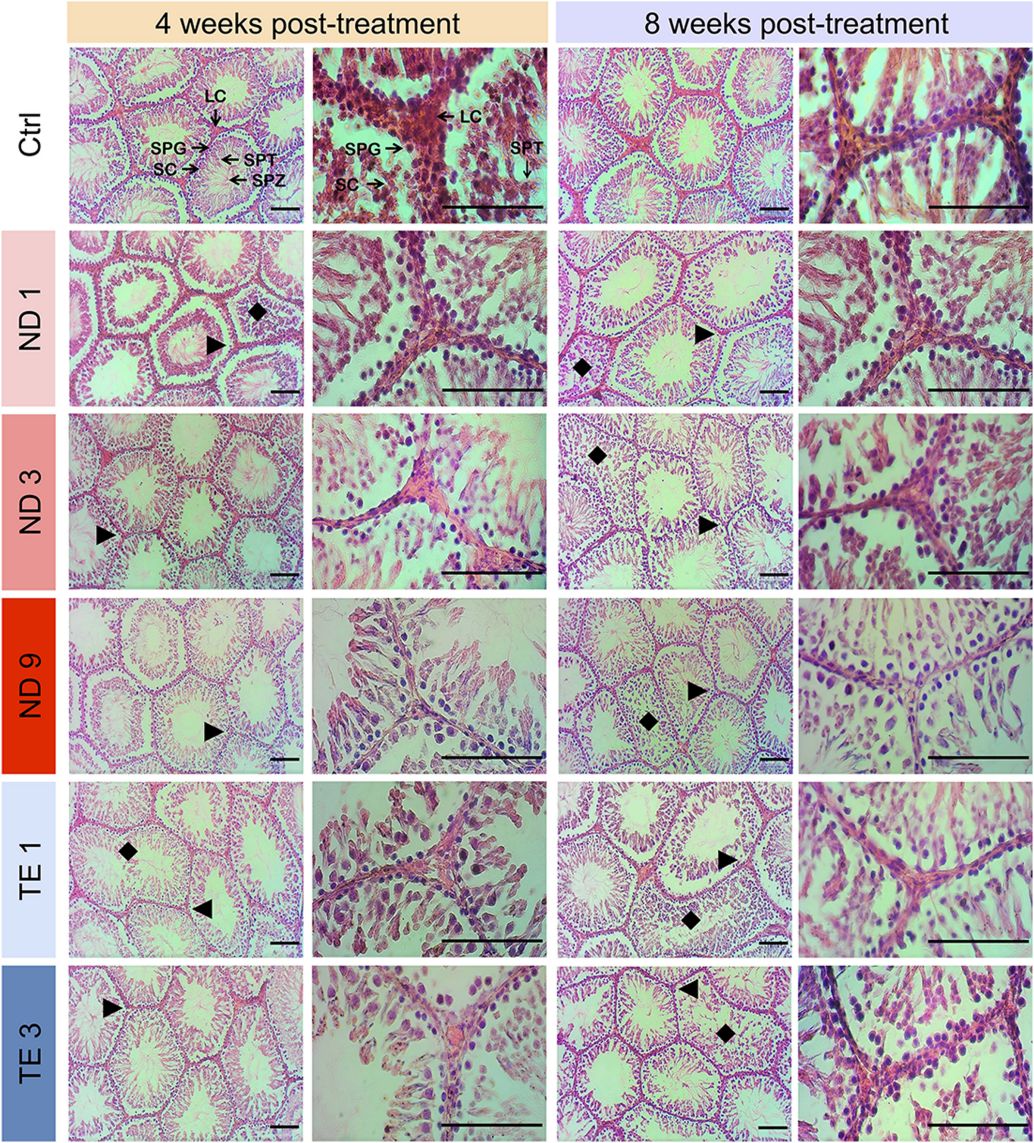
Nandrolone decanoate (ND) and testosterone enanthate (TE) abuses cause histological defects in the testes of male Wistar rats. Animals were weekly administrated with intramuscular injections of 1, 3, and 9 mg/kg/weeks ND, and 1 and 3 mg/kg TE. The testes were sampled at 1 week after the fourth injection (4 weeks post‐treatment) and eighth injection (8 weeks post‐treatment), and fixed in 10% formalin solution. Histological sections were stained with hematoxylin and eosin (H & E). After treatments with ND or TE, seminiferous tubules showed various abnormalities including disorders in germ cells divisions and arrangements (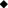), and sever depletion of the Leydig cells in the interstitial compartment (arrow heads). LC, Leydig cells; SPG, Spermatogonia; SC, Spermatocytes; SPT, Spermatids; SPZ, Spermatozoa. Scale bars are 100 μm.

**FIGURE 4:**
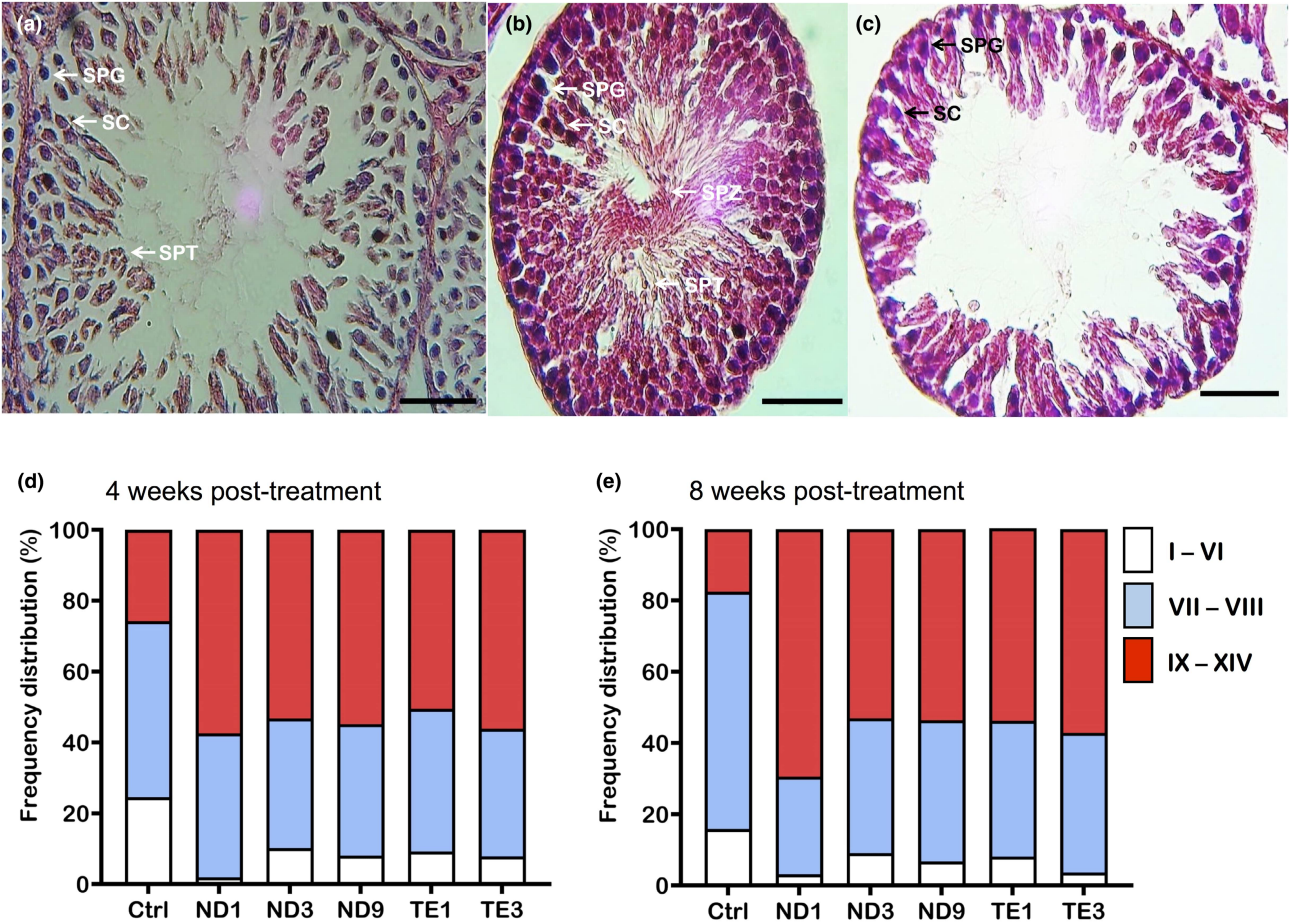
Abuses of nandrolone decanoate (ND) and testosterone enanthate (TE) delays spermatozoa development in male Wistar rats. Animals were weekly administrated with intramuscular injections of 1, 3, and 9 mg/kg ND and 1 and 3 mg/kg TE. The testes were sampled at 1 week after the fourth injection (4 weeks post‐treatment) and eighth injection (8 weeks post‐treatment), and fixed in 10% formalin solution. Histological sections were stained with hematoxylin and eosin (H & E), and cycles of seminiferous tubules were determined. Panels (a–c) are examples of Stages I–VI, VII–VIII, and IX–XIV of seminiferous cycle in the rats from control group sampled at 8 weeks post‐treatment, respectively. The seminiferous cycle was described in the section 2.4. Panels d and e show frequency of seminiferous tubules at Stages I–VI, VII–VIII, and IX–XIV at 4 and 8 weeks post‐treatment, respectively. SPG, Spermatogonia; SC, Spermatocytes; SPT, Spermatids; SPZ, Spermatozoa. Scale bars are 50 μm.

In the control group, seminiferous tubules were filled with spermatogonia, spermatocytes, spermatids, and spermatozoa indicating normal spermatogenesis (Figure 3). After treatment with ND or TE, seminiferous tubules showed various abnormalities including disorders in arrangement of germ cells and severe depletion of the Leydig cells in the interstitial compartment (Figure 3). Analysis of the seminiferous cycle (Figure 4a–c) showed lower and higher frequency of seminiferous tubules at Stages I–VI and stages IX–XIV, respectively, in ND or TE treated rats at 4 and 8 weeks post‐treatment (Figure 4d,e). The highest numbers of seminiferous tubules at Stages VII–VIII were seen in the control group at 4 and 8 weeks post‐treatment (Figure 4d,e). These results show a delay in spermatozoa development in rats treated with ND or TE.

Two‐way ANOVA showed a non‐significant AASs × treatment period interaction for the diameter of seminiferous tubules (Table 1; Figure S2A). Investigating the effects of main factors showed no significant effects of AASs or treatment period on the diameter of seminiferous tubules (Figure S2B,CC).

**FIGURE 5:**
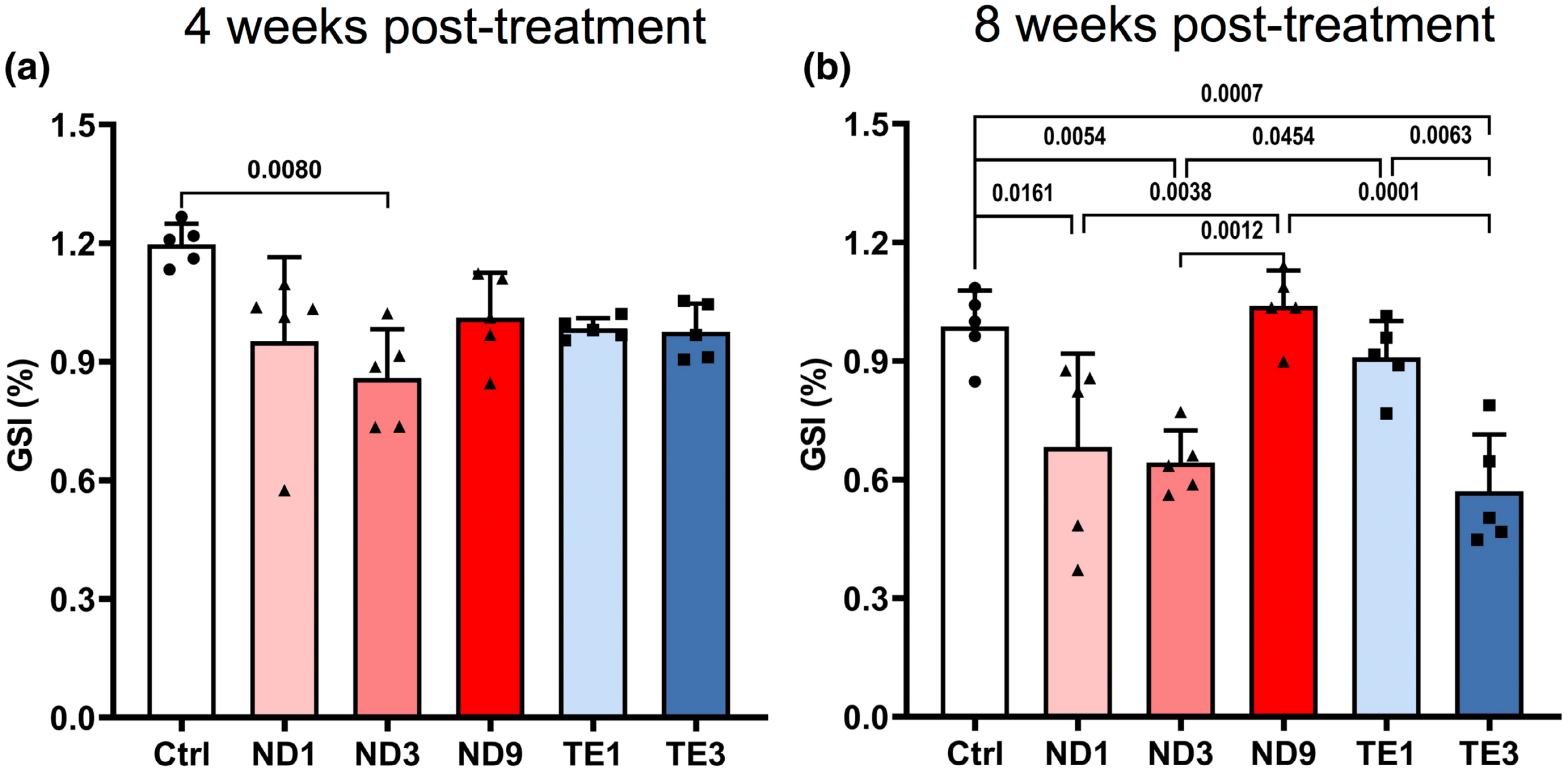
Nandrolone decanoate (ND) and testosterone enanthate (TE) abuses decrease gonadosomatic index (GSI) in the male Wistar rats. Animals were weekly administrated with intramuscular injections of 1, 3, and 9 mg/kg ND and 1 and 3 mg/kg TE. The testes were sampled to assess GSI (testes mass/body mass × 100) at 1 week after the fourth injection (4 weeks post‐treatment) and eighth injection (8 weeks post‐treatment). Two‐way ANOVA showed significant effects of AASs × treatment period interaction, thus the effect of AASs was investigated on GSI at 4 (a) and 8 (b) weeks post‐treatment using one‐way ANOVA followed by Tukey's multiple comparisons test. Data are shown as mean ± SD (*n* = 5). The actual *p* values are expressed.

Along with histological defects, two‐way ANOVA showed a significant AASs × treatment period interaction for GSI (*p* = 0.0337) (Table 1). Therefore, the model was revised to investigate the effects of AASs on GSI at each sampling time. At 4 weeks post‐treatment, GSI showed trends toward decreases in ND or TE groups compared to the control, which was significant in male rats that received 3 mg/kg/weeks ND (*p* = 0.0080) (Figure 5a). At 8 weeks post‐treatment, GSI was significantly decreased in rats administrated with 1 and 3 mg/kg/weeks ND and 3 mg/kg TE compared to the control group (Figure 5b). GSI of 9 mg/kg/weeks ND treated rats was similar to that of the control group (*p* = 0.9893), but it was higher than 1 and 3 mg/kg/weeks ND treated rats (*p* = 0.0038 and *p* = 0.0012, respectively). Also, GSI in 1 mg/kg/weeks TE treated rats was like that of the control group (*p* = 0.9376), but it was higher than GSI in 3 mg/kg/weeks TE treated rats (*p* = 0.0063).

### 3.3 Circulating T, E_**2**_ , T/E_2_ ratio, and LH levels

Two‐way ANOVA showed significant AASs × treatment period interactions for T, E_2_ and LH levels (Table 1). Therefore, the model was revised to investigate the effects of AASs on these hormones at each sampling time.

**FIGURE 6:**
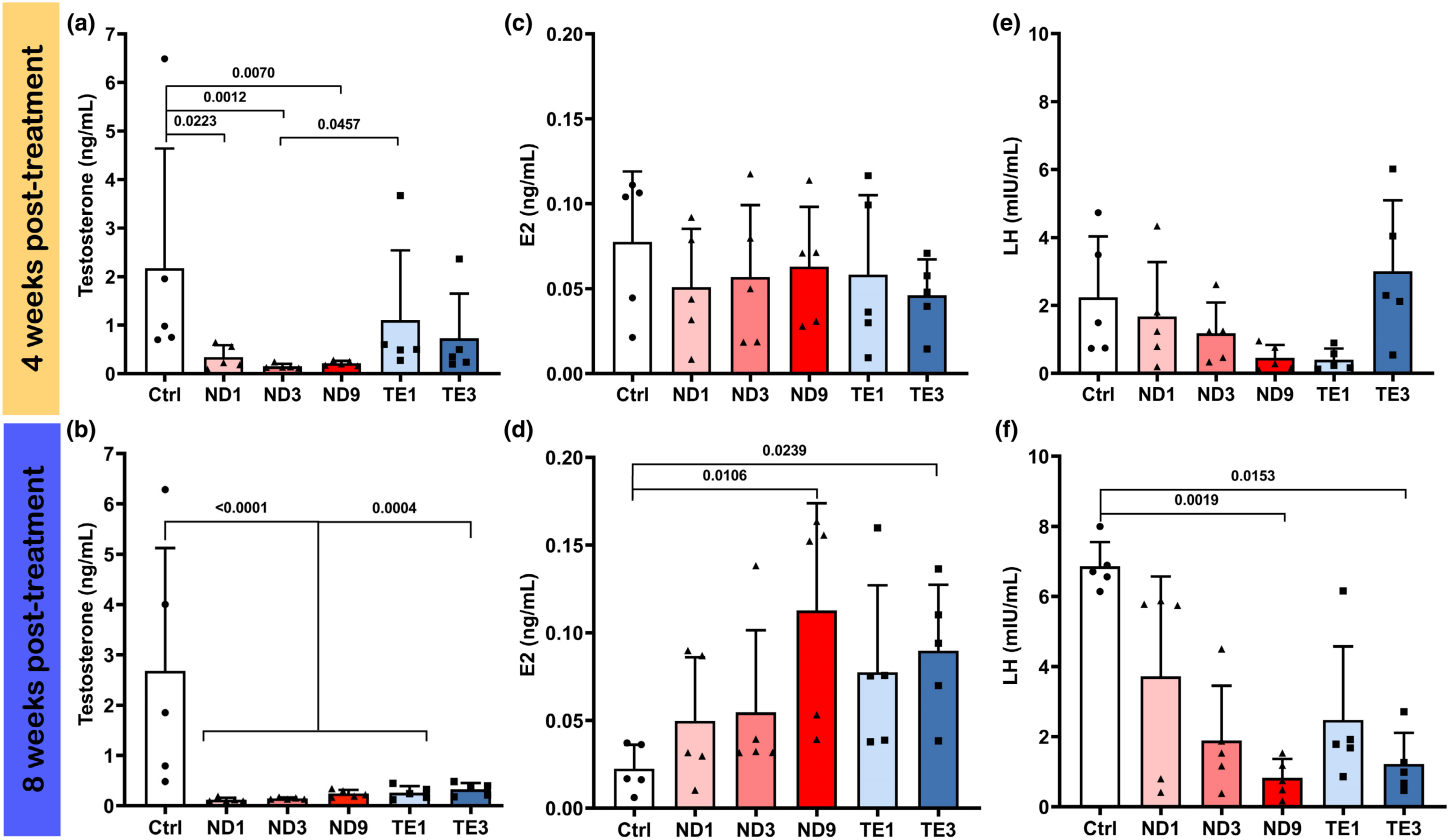
Nandrolone decanoate (ND) and testosterone enanthate (TE) abuses decrease circulating testosterone (T) and luteinizing hormone (LH) levels, and increase circulating 17β‐estradiol (E_2_) level in the male Wistar rats. Animals were weekly administrated with intramuscular injections of 1, 3, and 9 mg/kg ND and 1 and 3 mg/kg TE. Circulating T, E_2_, and LH were measured in the blood plasma using ELISA method at 1 week after the fourth injection (4 weeks post‐treatment) and eighth injection (8 weeks post‐treatment). Two‐way ANOVA showed significant effects of AASs × treatment period interaction, thus the effect of AASs was investigated on T, E_2_, and LH levels at 4 (a, c, and e) and 8 (b, d, and f) weeks post‐treatment using one‐way ANOVA followed by Tukey's multiple comparisons test. Data are shown as mean ± SD (*n* = 5). The actual *p* values are expressed.

At 4 weeks post‐treatment, T level was significantly decreased in rats that received ND compared to the control, while non‐significant decreasing trends were observed in TE treated rats (Figure 6a). Also, T level in 1 mg/kg/ weeks TE treated rats was higher than 3 mg/kg/ weeks ND treated rats (*p* = 0.0457). No significant changes in E_2_ (Figure 6c) and LH (Figure 6e) levels were observed among treatments. However, LH level showed decreasing trends in rats treated with 1, 3, and 9 mg/kg/ weeks ND and 1 mg/kg/ weeks TE.

At 8 weeks post‐treatment, administrations of ND and TE significantly decreased T level compared to the control group (Figure 6b). An increasing trend in E_2_ level was observed in ND and TE treated groups compared to the control, which were significant in rats treated with 9 mg/kg/ weeks ND (*p* = 0.0106) and with 3 mg/kg/ weeks TE (*p* = 0.0239) (Figure 6d). LH level showed decreasing trends in ND and TE treated rats, which were significant at 9 mg/kg/ weeks ND (*p* = 0.0019) and at 3 mg/kg/ weeks TE (*p* = 0.0153) (Figure 6f).

**FIGURE 7:**
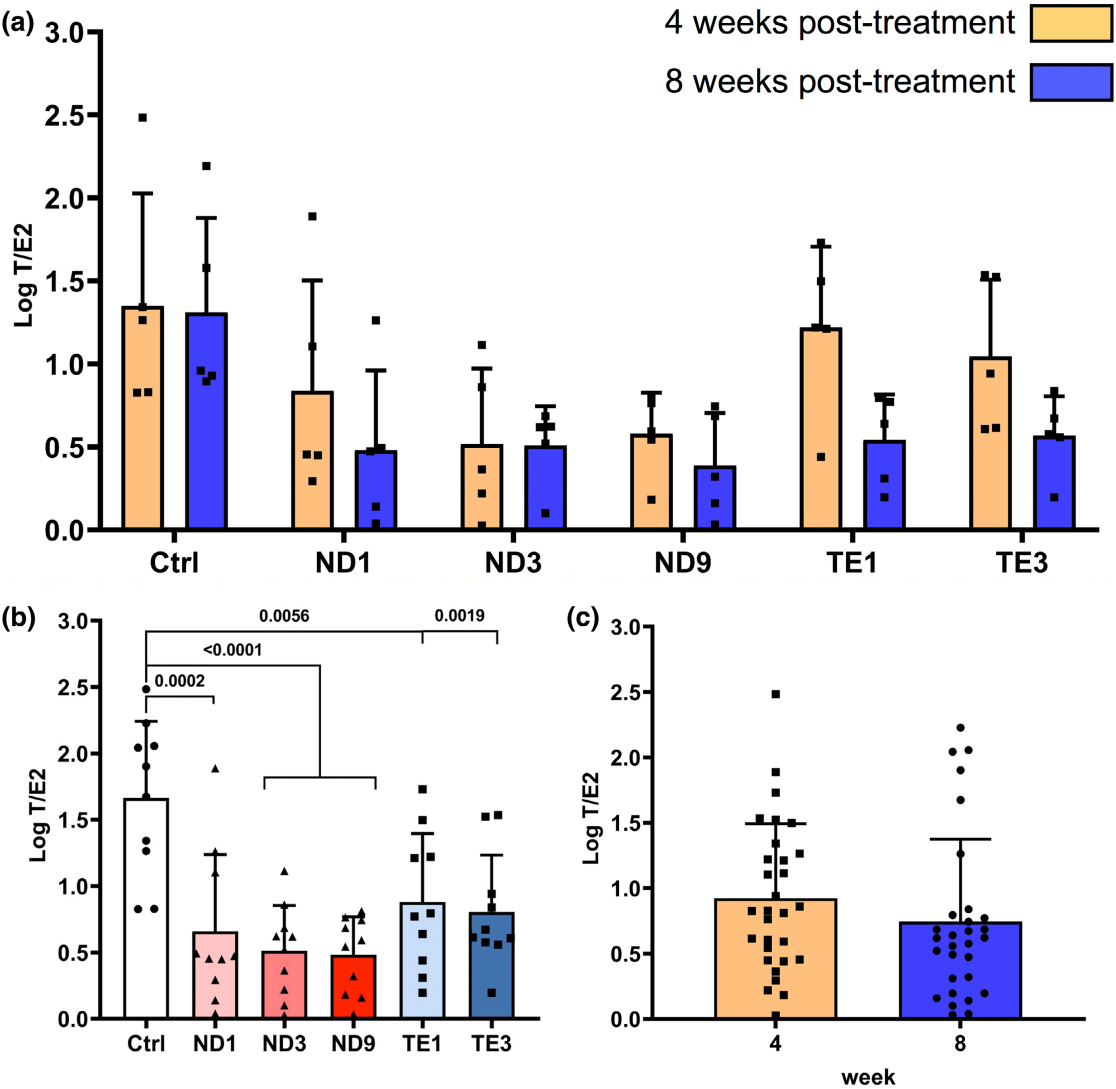
Nandrolone decanoate (ND) and testosterone enanthate (TE) abuses decrease testosterone (T)/17β‐estradiol (E_2_) ratio in the male Wistar rats. Animals were weekly administrated with intramuscular injections of 1, 3, and 9 mg/kg ND and 1 and 3 mg/kg TE. Circulating T and E_2_ were measured in the blood plasma using ELISA method at 1 week after the fourth injection (4 weeks post‐treatment) and eighth injection (8 weeks post‐treatment). Two‐way ANOVA showed non‐significant effects of AASs × treatment period interaction (a), thus the model was revised to study the effects of AASs (b) and treatment period (c) on T/E_2_ ratio using one‐way ANOVA followed by Tukey's multiple comparisons test and independent *t*‐test, respectively. Data are shown as mean ± SD (*n* = 5). The actual *p* values are expressed.

Regarding T/E_2_ ratio, the main effects were studied due to a non‐significant AASs × treatment period interaction (Table 1; Figure 7a). When the effects of AASs were studied, compared to the control, T/E_2_ ratio was decreased in both ND (*p* = 0.0002 or *p* < 0.0001) and TE treated rats (*p* = 0.0056 and *p* = 0.0019) (Figure 7b). However, T/E_2_ ratio was not differed between 4 and 8 weeks post‐treatments (Figure 7c).

## 4 DISCUSSION

It has been demonstrated that AASs abuse induces hypogonadotropic hypogonadism (Table S1). The present study was designed to assess ND‐induced hypogonadotropic hypogonadism in a dose‐ and time‐dependent manner in conjunction with impairments in testicular histology. Male Wistar rats were administrated with 1, 3, and 9 mg/kg/weeks ND for 8 weeks, and body gain, HSI, GSI, and circulating T, E_2_, T/E_2_ ratio and LH levels were assessed at 4 and 8 weeks post‐treatment. To better understand the effects of ND abuse on reproductive system, groups of rats were treated with 1 and 3 mg/kg/weeks TE. Results showed ND and TE reduction of GSI and inhibition of T biosynthesis at 4 and 8 weeks post‐treatment, respectively. In ND treated rats, a significant decrease in GSI was observed along with decrease in T level, however trends toward decrease in LH level showed no significant differences. In TE treated rats, GSI was decreased along with decrease in both T and LH levels suggesting a hypogonadotropic hypogonadism. Interestingly, we observed that abuse of ND at therapeutic dose (1 mg/kg/ weeks) caused hypogonadism. These show that abuse of both ND and TE interferes with testes or pituitary functions to cause hypogonadism along with disruption of spermatogenesis. However, the adverse effects of ND and TE depend on dose and treatment period.

In the present study, sample size was five for each treatment, and treated rats were without exercise after administration of AASs. We also used similar protocol in further experiments to study the effects of AASs abuse on fertility and their epigenetic effects in the next generation. Results showed induction of hypogonadism with similar disruptions of pituitary and testicular hormonal functions in both non‐exercised and exercised rats administrated with similar doses of ND (1, 3, and 9 mg/kg/weeks) and TE (1 and 3 mg/kg/weeks) (Kazori, 2022). When AASs are used to treat patients for clinical purpose, AASs receivers are without exercise, and also non‐athletes AASs abusers may have no exercise. Moreover, previous studies have shown similar alternations of reproductive function in exercised and non‐exercised animal models treated with AASs including ND (Barone et al., 2017; Naraghi et al., 2010; Selakovic et al., 2019; Shokri et al., 2014). On the other hand, abuse of AASs disrupts reproductive system irrespective to physical activity or exercise.

We observed that administrations of ND resulted in a GSI decrease in rats that received 3 and 1 mg/kg/weeks ND at 4 and 8 weeks post‐treatment, respectively, however a higher dose of ND was without effect on GSI as reported by Alves et al. (2024). These results suggest that (I) ND is capable of inducing hypogonadism at low dose, and (II) the low‐dose ND induced hypogonadism occurs when the period of treatment is longer. Different from ND, decrease in GSI was not observed at 1 mg/kg/ weeks TE at 8 weeks post‐treatment, suggesting different potencies compared to ND to cause hypogonadism. In contrast to our observation, ND induced hypogonadism at higher doses have been reported: 10 mg/kg/weeks at 4 or 8 weeks post‐treatment (Ahmed, 2015; Saddick, 2021; Shokri et al., 2010) and 30 g/kg/weeks at 12 weeks post‐treatment (Kahal & Allem, 2018). Moreover, Grönbladh et al. (2013) reported increase in testicular mass in adult rats administrated with subcutaneous injection of 15 mg/kg ND every 3 days for 3 weeks. However, boldenone undecylenate and testosterone undecanoate (TU) have been reported to cause hypogonadism at similar dose and treatment time (Behairy et al., 2020; Oda & El‐Ashmawy, 2012). These controversies among studies might be related to dose of AASs and duration of treatment. It is worth to note that ND‐induced hypogonadism was observed in both exercised and non‐exercised rats (Shokri et al., 2010).

The effects of ND and TE on GSI were along with pathological effects and delay in the seminiferous cycle. We observed disorganization in seminiferous tubule and lack or atrophy of Leydig cells in interstitial compartment supporting AAS‐induced diminished spermatozoa production reported in previous studies (Table S1). Similar histopathological defects have been previously reported as well as elimination of spermatogenesis (Abed et al., 2022; Ahmed, 2015; Al‐Otaibi, 2024; Alves et al., 2024; Barone et al., 2017; Behairy et al., 2020; Jannatifar et al., 2015; Kahal & Allem, 2018; Mesbah et al., 2008; Naraghi et al., 2010; Saddick, 2021; Takahashi et al., 2004) that might be due to induction of apoptosis and testicular oxidative damage (Ahmed, 2015; Behairy et al., 2020). The present study is the first that shows the effects of AASs on the seminiferous cycle suggesting a delay in spermatogenesis as numbers of seminiferous at Stages IX–XIV were higher in ND or TE treated rats. However, ND and TE were without effects on diameter of seminiferous tubules as reported by Alves et al. (2024). The AASs effects on diameter of seminiferous tubules are controversial among studies that might be due to dose and duration of treatment or the shape of seminiferous used to determine its diameter (Ahmed, 2015; Saddick, 2021).

In male, T and balance of androgens to estrogens are essential for reproductive development and spermatogenesis (Smith & Walker, 2015; Williams et al., 2001). The present study showed that circulating T level was significantly decreased in all ND treated groups at 4 weeks post‐treatment and maintained significantly lower than T level in the control rats at 8 weeks post‐treatment. Compared to ND, significant decreases in T level were observed at 8 weeks post‐treatment in TE treated rats. These suggest that both ND and TE are capable of inhibiting T biosynthesis, however their effects depend on duration of treatment. The ND or TE inhibition of T biosynthesis might be related to lack, atrophy or apoptosis of Leydig cells (Mesbah et al., 2008; Saddick, 2021) associated with down‐regulation of steroidogenic enzymes (Alsiö et al., 2009; Koeva et al., 2003; Min & Lee, 2018). We observed that significant decrease in GSI was associated with significant reduction of circulating T level in both ND and TE treated rats. Our results are consistent with previous studies, and suggest that AAS‐induced hypogonadism is accompanied by inhibition of T biosynthesis (Al‐Otaibi, 2024; Alves et al., 2024; Abed et al., 2022; Ibrahim et al., 2022; Behairy et al., 2020; Barone et al., 2017; Ahmed, 2015; Jannatifar et al., 2015; Mohamed & Mohamed, 2015; Grönbladh et al., 2013; Oda & El‐Ashmawy, 2012; Breuer et al., 2001). However, there are studies that report elevation of circulating T level in rats received AASs including ND (Frankenfeld et al., 2014; Selakovic et al., 2017), TE (Ai et al., 2007; Selakovic et al., 2019) and TU (Zhang et al., 2016) and testosterone propionate (TP) (Breuer et al., 2001). These might be due to metabolism of T esters (such as TE, TU, and TP) to T, which may result in higher plasma T level. The controversy among studies might be related to age of animal models at the time of first AASs administration, route of AASs administration, dose of AASs and duration of treatment. For instance, Selakovic et al. (2017 and 2019) subcutaneously administrated 20 mg/kg/weeks ND or TE to 3‐month‐old rats for 6 weeks. To support AAS induction of T biosynthesis, there is also a study showing that nandrolone at 3.9 μM (equal to 0.97 mg/L) is capable of inducing T biosynthesis in the Leydig cells, in vitro (Pomara et al., 2016).

Studies that assessed circulating LH level suggested AAS‐induced hypogonadotropic hypogonadism (Behairy et al., 2020; Breuer et al., 2001; Ibrahim et al., 2022; Shahraki et al., 2015; Sretenovic et al., 2020). In the present study, among the experimental groups showing hypogonadism, LH level was only decreased in rats administrated with 3 mg/kg/ weeks TE, significantly. Among ND treatments, LH level was significantly decreased in rats administrated with 9 mg/kg/ weeks ND. Observed trends toward decreases in LH levels were not significant in rats administrated with 1 and 3 mg/kg/ weeks ND due to inter‐animal variations in LH levels. Generally, data show that AAS abuse was along with decrease in circulating LH level, and suggest that AAS inhibition of T biosynthesis could not activate the negative feedback loop to stimulate LH biosynthesis or release from pituitary. In this context, Sretenovic et al. (2020) reported decreases in the volume density and number of LH and FSH cells in the pituitary of rats received 20 mg/kg/weeks ND for 4 weeks.

It has been suggested that administrations of AASs result in elevation of circulating E_2_ level (Grönbladh et al., 2013; Selakovic et al., 2017; Takahashi et al., 2004). Our results also show significant increase in E_2_ level in rats treated with 9 mg/kg/weeks ND and with 3 mg/kg/ weeks TE at 8 weeks post‐treatment. However, decrease in T level and increase in E_2_ level resulted in imbalance between estrogens and androgens that is prerequisite for reproductive development and functions (Williams et al., 2001). Therefore, lower GSI and histological defects in the testes of rats treated with ND and TE might be due to remarkable decrease in T/E_2_ ratio. This is supported by the fact that increase in E_2_ level resulting in decrease of T/E_2_ ratio was along with up regulation of cytochrome P450 aromatase activity (Quignot et al., 2012).

Despite ND and TE effects on the reproductive system, we observed that weight gain did not differ between 4 and 8 weeks post‐treatment. Our observation is different from previous studies showing AAS inhibition of weight gain along with decreases in food and water intake (Beutel et al., 2005; Kahal & Allem, 2018; Long et al., 2000; Min & Lee, 2018) that might be due to AAS dose. We observed no significant difference in HSI among treatments, however it has been shown that administration of AASs may reduce hepatocyte numbers and cause liver neoplasia (Petrovic et al., 2022; Sánchez‐Osorio et al., 2008).

## 5 CONCLUSIONS AND FUTURE PERSPECTIVES

In conclusion, these results indicate that abuse of ND and TE inhibits T biosynthesis resulting in hypogonadism in male rats and affect testicular compartment. We observed that abuse of ND at therapeutic dose (1 mg/kg/ weeks) also causes hypogonadism. Although, LH levels were decreased in both ND and TE treated rats, significant differences were noted for 9 mg/kg/ weeks ND and for 3 mg/kg/weeks TE. This decrease in circulating LH level suggests that the negative feedback of T was disrupted in ND and TE treated rats. Comparing the effects of ND and TE at the same concentrations and treatment period reveals different potencies in causing hypogonadism or interfering with hormonal functions of testes and pituitary. Future research needs to utilize transgenic animal models with deficient AR to elucidate AASs disruption of T signaling in reproduction. The hypothalamic regulation of pituitary and testicular functions should be investigated to uncover AASs modes of action on reproductive system. In addition, studying sperm motility, microRNA and epigenome provide valuable information to understand AASs consequences on fertility and their transgenerational inheritance.

## AUTHOR CONTRIBUTION

*Conceptualization*: S.M.H.A., S.K., and N.K. *Investigation*: S.K., N.K., S.A., M.A.A.S., and S.M.H.A. *Data curation*: S.K., N.K., S.A., and S.M.H.A. *Formal analysis*: S.K., S.A., N.K., M.A.A.S., and S.M.H.A. *Methodology*: N.K., S.M.H.A., and B.Z. *Funding acquisition*: S.M.H.A. *Project administration*: S.M.H.A. *Supervision*: S.M.H.A. and B.Z. *Resources*: S.M.H.A. and B.Z. *Writing–manuscript draft*: N.K., S.K., and S.M.H.A. *Writing*–*review and editing*: N.K. and S.M.H.A. All authors contributed to the article and approved the submitted version.

## FUNDING INFORMATION

None.

## CONFLICT OF INTEREST STATEMENT

The authors declare no conflict of interest.

## ETHICS STATEMENT

All animal experiments comply with Animal Research: Reporting of In Vivo Experiments (ARRIVE) guidelines (Percie du Sert et al., 2020) and the NIH‐National Research Council Guide for the Care and Use of Laboratory Animals (National Research Council, 2011). Experimental protocols and procedures were approved by the Council for Research and Graduate Studies of the College of Science according the Central Ethics Committee for management, realization, and control of the experiments on animals at UT (397857/K6‐05). SMHA, SK, NK, and SA owned the certificates of professional competence, completed appropriate training for maintenance and handling of the experimental animals. This experiment was performed under direct control and supervision of the coordinator of Animal Research Laboratory at IBB, UT.

## Supporting information


**Figure S1.** Nandrolone decanoate (ND) and testosterone enanthate (TE) abuses do not affect hepatosomatic index (HSI) in the male Wistar rats (A). Animals were weekly administrated by intramuscular injections of 1, 3, and 9 mg/kg ND and 1 and 3 mg/kg TE. The liver was sampled to assess HSI (Liver mass/body mass × 100) at 1 week after the fourth injection (4 weeks post‐treatment) and 8th injection (8 weeks post‐treatment). Two‐way ANOVA showed non‐significant effects of AASs × treatment period interaction (A), thus model were revised to study the effects of AASs (B) and treatment period (C) on HSI using one‐way ANOVA followed by Tukey’s post hoc test and independent *t*‐test, respectively. Data are shown as mean ± SD (*n* = 5). The actual *p* values are expressed.

**Figure S2.** Nandrolone decanoate (ND) and testosterone enanthate (TE) abuses do not affect diameter of seminiferous tubules (ST) in the male Wistar rats (A). Animals were weekly administrated by intramuscular injections of 1, 3, and 9 mg/kg ND and 1 and 3 mg/kg TE. The testes were sampled at 1 week after the fourth injection (4 weeks post‐treatment) and 8th injection (8 weeks post‐treatment), and fixed in 10% formalin solution. Histological sections were stained with H & E, and diameter of ST was measured. Two‐way ANOVA showed non‐significant effects of AASs × treatment period interaction (A), thus model were revised to study the effects of AASs (B) and treatment period (C) on ST diameter using one‐way ANOVA followed by Tukey’s multiple comparisons test and independent *t*‐test, respectively. Data are shown as mean ± SD (*n* = 5).

Data S1.

## Data Availability

The data supporting the study's findings are provided in the publication and its supplementary materials.
