# Supplementary figures and images for: Nandrolone decanoate–induced hypogonadism in male rats: Dose‐ and time‐dependent effects on pituitary and testicular hormonal functions
Source: Physiol Rep. 2024 Oct 6;12(19):e70053. doi: 10.14814/phy2.70053 (PMC11456365; doi:10.14814/phy2.70053)

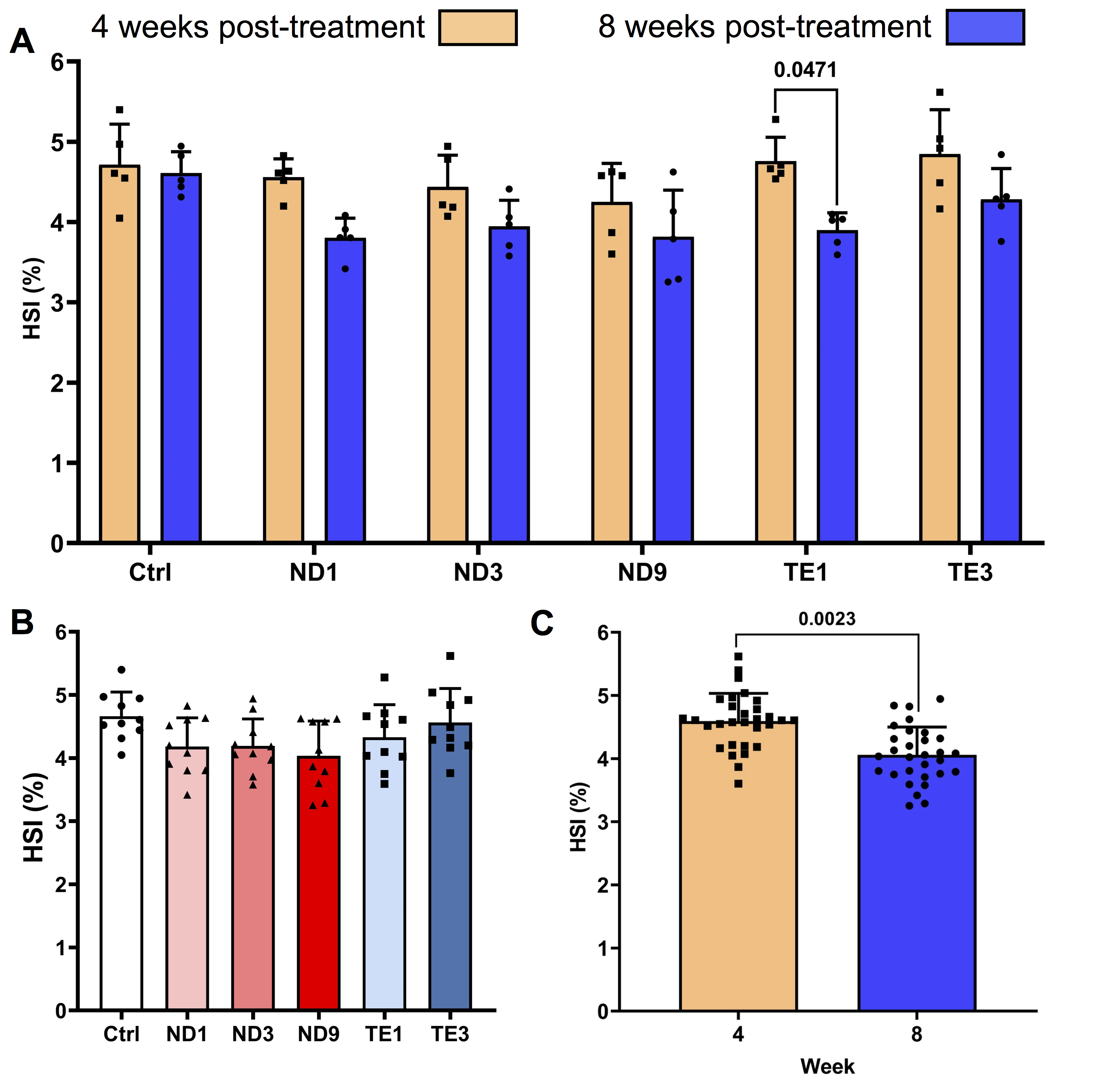

Supplement: Supplementary file 1 — Figure S1. Nandrolone decanoate (ND) and testosterone enanthate (TE) abuses do not affect hepatosomatic index (HSI) in the male Wistar rats (A). Animals were weekly administrated by intramuscular injections of 1, 3, and 9 mg/kg ND and 1 and 3 mg/kg TE. The liver was sampled to assess HSI (Liver mass/body mass × 100) at 1 week after the fourth injection (4 weeks post‐treatment) and 8th injection (8 weeks post‐treatment). Two‐way ANOVA showed non‐significant effects of AASs × treatment period interaction (A), thus model were revised to study the effects of AASs (B) and treatment period (C) on HSI using one‐way ANOVA followed by Tukey’s post hoc test and independent t‐test, respectively. Data are shown as mean ± SD (n = 5). The actual p values are expressed. [file PHY2-12-e70053-s002.jpg]

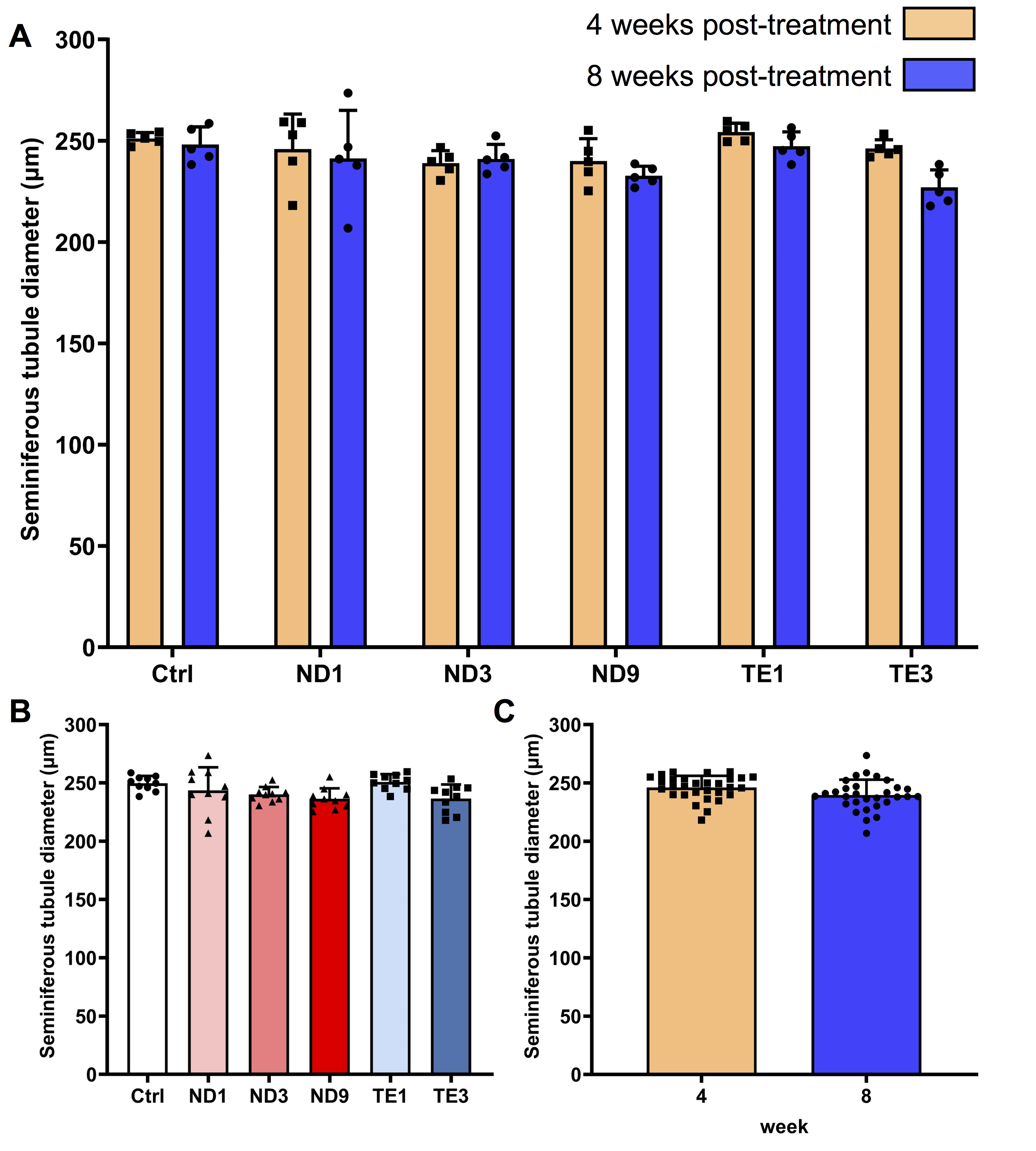

Supplement: Supplementary file 2 — Figure S2. Nandrolone decanoate (ND) and testosterone enanthate (TE) abuses do not affect diameter of seminiferous tubules (ST) in the male Wistar rats (A). Animals were weekly administrated by intramuscular injections of 1, 3, and 9 mg/kg ND and 1 and 3 mg/kg TE. The testes were sampled at 1 week after the fourth injection (4 weeks post‐treatment) and 8th injection (8 weeks post‐treatment), and fixed in 10% formalin solution. Histological sections were stained with H & E, and diameter of ST was measured. Two‐way ANOVA showed non‐significant effects of AASs × treatment period interaction (A), thus model were revised to study the effects of AASs (B) and treatment period (C) on ST diameter using one‐way ANOVA followed by Tukey’s multiple comparisons test and independent t‐test, respectively. Data are shown as mean ± SD (n = 5). [file PHY2-12-e70053-s001.jpg]
