# Supplementary material for: Nandrolone decanoate–induced hypogonadism in male rats: Dose‐ and time‐dependent effects on pituitary and testicular hormonal functions
Source: Physiol Rep. 2024 Oct 6;12(19):e70053. doi: 10.14814/phy2.70053 (PMC11456365; doi:10.14814/phy2.70053)
Supplement: Supplementary file 3 — Data S1. [file PHY2-12-e70053-s003.docx]

**Supplementary materials - Tables**

**Nandrolone decanoate–induced hypogonadism in male rats: Dose- and time-dependent effects on pituitary and testicular hormonal functions**

Sholeh Karimi ^¶^, Negar Kazori ^¶^, Sayyed Mohammad Hadi Alavi ^*^, **Sara Alijanpour,** Mohammad Alim Atif Siddiqi †, **Bahman Zeynali**

Department of Animal Biology, School of Biology, College of Science, University of Tehran, Tehran 14176-14411, Iran

^¶^ These authors contributed equally to this work as first author.

† Current address: Department of Biology, Faculty of Education, Loger Higher Education Institute, Loger, Afghanistan

* Corresponding author: S.M.H. Alavi, E-mail: [hadi.alavi@ut.ac.ir](mailto:hadi.alavi@ut.ac.ir)

**Table S1.** A summary of selected studies investigated the adverse effects of anabolic-androgenic steroids (AASs) abuses on reproductive system. Significant increase and decrease are shown by “↑” and “↓”, respectively.

| **Model** | **AAS** | **Dose**  **(mg/kg)** | **Administration** | **n** | **Exercise** | **Endpoints** | | | | | | | | **References** |
| --- | --- | --- | --- | --- | --- | --- | --- | --- | --- | --- | --- | --- | --- | --- |
|  |  |  |  |  |  | **Weight**  **Gain** | **GSI** | **HSI** | **Histology defects** | **T** | **E2** | **LH** | **Sperm** |  |
| rat | Trenorol | 10 | 4 W, weekly | 10 | × |  | ↓ |  | Irregular germ cells arrangement  Decrease in spermatogenic cells  Leydig cells lost | ↓ |  | ↓ | ↓ count  ↓ motility  ↓ viability  ↑ Abnormal  morphology | Al-Otaibi, 2024 |
| rat | ND | 1 | 8 w, weekly | 6 | × | ns | ↓ | ns | Irregular germ cells arrangement  Empty lumen  Decrease in seminiferous tubules diameter and germinal epithelium height  Spermatogenic arrest | ↓ | ns | ns |  | Kazori, 2022 |
|  |  | 3 |  |  |  |  | ↓ | ns |  | ↓ | ns | ↓ |  |  |
|  |  | 9 |  |  |  |  | ns | ns |  | ↓ | ↑ | ↓ |  |  |
| rat | ND | 10 | 8 w, weekly | 8 | × | ns | ns |  |  | ↓ |  |  | ↓ count  ↓ viability  ↑ Abnormal  morphology | Alves et al., 2024 |
| rat | Oxandrolone | 0.28 | 14D, daily | 7 | × | ns | ↓ |  |  | ↓ |  |  | ↓ count | Abed et al., 2022 |
| rat | ND | 2.5 | 10 w, weekly |  | × |  |  |  | Irregular germ cells arrangement  Empty lumen |  |  |  |  | Ibrahim et al., 2022 |
|  |  | 5 |  | 10 |  | ↓ |  |  |  |  |  |  |  |  |
|  |  | 10 |  |  |  | ↓ |  |  |  | ↓ |  | ↓ |  |  |
| rat | ND | 10 | 4 w, weekly | 10 (ctrl)  18 (ND) | × |  | ns |  | Leydig and spermatogenic cells apoptosis |  |  |  | ↑ apoptosis | Saddick, 2021 |
| rat | BOL | 5 | 8 w, weekly | 8 | × | ↑ | ↓ |  | Decrease in spermatocytes and spermatids | ↑#  ↓* | ↓ | ↓ | ↓ count  ↓ motility  ↑ Abnormal  morphology | Behairy et al., 2020 |
| rat | ND | 20 | 6 w, weekly | 8 | × |  |  |  |  | ↑ | ↑ |  |  | Selakovic et al., 2017, 2019 |
|  |  |  |  |  | 5 d/w |  |  |  |  | ↑ | ↑ |  |  |  |
|  | TE |  |  |  | × |  |  |  |  | ↑ | ns |  |  |  |
|  |  |  |  |  | 5 d/w |  |  |  |  | ↑ | ns |  |  |  |
| mice | ND | 10 | 6 w, twice/w | 6 | × |  |  |  | Seminiferous disorganization  Leydig cells and spermatids lost | ↓ |  |  |  | Barone, et al., 2017 |
|  |  |  |  |  | 5 d/w |  |  |  | ns | ↓ |  |  |  |  |
|  |  | 20 |  |  | × |  |  |  | Seminiferous disorganization  Leydig cells and Spermatids lost | ↓ |  |  |  |  |
|  |  |  |  |  | 5 d/w |  |  |  |  |  |  |  |  |  |
| rat | TU | 45 | 12 w, every 4 w | 30 | × |  | ↓ |  |  |  |  |  | ↓ Fertility  ↓ count  ↓ motility  ↑ Abnormal  morphology | Zhang et al., 2016 |
| rat | ND | 3 | 8 w, weekly | 6 | × |  |  |  |  | ns |  |  | ↓ count  ↓ motility | Mohamed and Mohamed, 2015 |
|  |  | 10 |  |  |  |  |  |  |  | ↓* |  |  |  |  |
| rat | ND | 10 | 10 w  weekly | 10 |  |  | ↓ |  |  |  |  |  | ↓ Fertility  ↓ count  ↓ motility  ↑ Abnormal  morphology | Shokri et al., 2010, 2014 |
|  |  |  |  |  | ×  5 d/w |  | ↓ |  |  |  |  |  |  |  |
| rat | ND | 15 | 3 w  every 3 days | 12 | × | ↓ | ↑ | ns |  | ↓ | ↑ |  |  | Grönbladh et al., 2013 |
| rabbit | BOL | 4.4 | 8 w  twice per week | 10 | × |  | ↓ |  | Seminiferous disorganization | ↓ |  |  | ↓ volume  ↓ count  ↓ motility  ↑ Abnormal  morphology | Oda and El-Ashmawy, 2012 |
|  |  | 8.8 |  |  |  |  | ↓ |  |  | ↓ |  |  |  |  |
| rat | ND | 10 | 8 w  weekly | 7 | × |  |  |  | spermatogenic cells apoptosis |  |  |  |  | Naraghi et al., 2010 |
|  |  |  |  |  | 5 d/w |  |  |  |  |  |  |  |  |  |
| rat | ND | 3 | 2 w  daily | 10 | × | ns |  |  |  |  |  | ↓ LHβ mRNA |  | Alsiö et al., 2009 |
|  |  | 15 |  |  |  | ↓ |  |  |  |  |  |  |  |  |
| rat | ND | 3 | 14 w  weekly | 30 | × |  |  |  |  |  |  |  | ↓ count  ↓ motility  ↑ Abnormal  morphology | Karbalay-Doust et al., 2007 |
|  |  | 10 |  |  |  |  |  |  |  |  |  |  |  |  |
| rat | TP | 5 | 12 w  5 d/w | 12 | × | ns | ↓ |  |  | ↑ |  | ↓ |  | Breuer et al., 2001 |
|  | ND |  |  |  |  |  |  |  |  | ↓ |  |  |  |  |

“×” shows studies or treatments without exercise after administration of AASs. “ns“ shows no significant effects was observed. “#” and “*” Shows serum and testicular T, respectively. Empty spaces for endpoints show no assessment.

For Barone et al., 2017, ND at 3.75 mg/kg was without effects on T level, and histology of the testes was similar to those of control.

Abbreviations of AASs: Nandrolone decanoate, ND; Boldenone undecylenate, BOL; Testosterone enanthate, TE; Testosterone undecanoate, TU; Testosterone propionate, TP; Testosterone, T.

**Table S2.** Body weight mass (g) of the male Wistar rats during 8 weeks of the experiment. Nandrolone decanoate (ND) and testosterone enanthate (TE) were intramuscularly injected once a week for 8 weeks. Data are shown as mean ± SD (*n* = 5).

|  | Dose  mg/kg/w | Weeks post-treatment | | | | | | | | |
| --- | --- | --- | --- | --- | --- | --- | --- | --- | --- | --- |
|  |  | Injection time | 1 | 2 | 3 | 4 (Sampling) | 5 | 6 | 7 | 8 (Sampling) |
|  |  | These groups were sampled at one week after the 4^th^ injection (4 weeks post-treatment) (*n* = 5 per group). | | | | | | | | |
| Ctrl |  | 218.8 ± 35.3 | n.d. | 235.2 ± 32.0 | 247.0 ± 24.5 | 255.8 ± 28.9 |  |  |  |  |
| ND | 1 | 224.0 ± 35.4 | n.d. | 243.6 ± 31.8 | 254.6 ± 32.7 | 264.4 ± 37.0 |  |  |  |  |
| ND | 3 | 211.8 ± 54.00 | n.d. | 244.2 ± 41.7 | 261.4 ± 41.6 | 279.0 ± 43.9 |  |  |  |  |
| ND | 9 | 220.6 ± 32.7 | n.d. | 244.0 ± 35.8 | 254.4 ± 30.5 | 264.4 ± 34.3 |  |  |  |  |
| TE | 1 | 218.4 ± 9.6 | n.d. | 247.0 ± 19.5 | 265.8 ± 19.6 | 280.0 ± 18.7 |  |  |  |  |
| TE | 3 | 213.8 ± 9.7 | n.d. | 238.4 ± 7.0 | 250.6 ± 10.4 | 253.2 ± 13.2 |  |  |  |  |
|  |  | These groups were sampled at one week after the 8^th^ injection (8 weeks post-treatment) (*n* = 5 per group). | | | | | | | | |
| Ctrl |  | 253.0 ± 18.6 | n.d. | 281.6 ± 18.4 | 286.6 ± 17.1 | 299.9 ± 22.8 | 308.7 ± 22.1 | 318.2 ± 23.4 | 327.9 ± 21.0 | 334.2 ± 23.6 |
| ND | 1 | 206.0 ± 54.4 | n.d. | 231.4 ± 44.8 | 236.0 ± 44.1 | 248.4 ± 51.9 | 260.0 ± 54.5 | 261.2 ± 61.9 | 267.7 ± 59.2 | 270.0 ± 61.0 |
| ND | 3 | 222.4 ± 49.4 | n.d. | 239.4 ± 40.3 | 259.0 ± 31.8 | 270.2 ± 31.3 | 277.0 ± 28.8 | 290.0 ± 23.8 | 298.8 ± 22.6 | 304.0 ± 25.6 |
| ND | 9 | 213.6 ± 31.2 | n.d. | 230.8 ± 29.7 | 242.0 ± 29.1 | 247.0 ± 33.3 | 257.0 ± 31.7 | 253.6 ± 33.4 | 255.4 ± 31.2 | 259.8 ± 29.2 |
| TE | 1 | 226.2 ± 8.5 | n.d. | 243.2 ± 5.4 | 251.8 ± 9.2 | 260.4 ± 13.0 | 268.8 ± 15.1 | 285.2 ± 18.6 | 293.0 ± 21.0 | 295.6 ± 21.8 |
| TE | 3 | 218.8 ± 21.4 | n.d. | 230.8 ± 21.1 | 240.0 ± 21.8 | 248.3 ± 21.9 | 256.0 ± 22.6 | 265.8 ± 22.1 | 276.0 ± 22.4 | 275.6 ± 16.9 |

**References**

Abed, A. F., Jarrar, Y. B., Al-Ameer, H. J., Al-Awaida, W., & Lee, S.-J. (2022). The protective effect of metformin against Oxandrolone-induced infertility in male rats. *Current Pharmaceutical Design, 28(4),* 324–330.

Al-Otaibi, A. M. (2024). Therapeutic effects of vitamin B17 against anabolic steroid Trenorol induced testicular toxicity, injury, DNA damage and apoptosis in male rats. *Toxicology Research, 13(2),* tfae084.

Alsiö, J., Birgner, C., Björkblom, L., Isaksson, P., Bergström, L., Schiöth, H. B., & Lindblom, J. (2009). Impact of nandrolone decanoate on gene expression in endocrine systems related to the adverse effects of anabolic androgenic steroids. *Basic & Clinical Pharmacology & Toxicology, 105(5),* 307–314.

Alves, F. L., Oliveira, M. A. F., de Morais, A. N. P., Martins, S. D., de Sá, N. A. R., Ceccatto, V. M., Watanabe, Y. F., & Araújo, V. R. (2024). Supraphysiological doses of nandrolone decanoate disrupts spermatogenesis but did not interfere on embryo rate. *Naunyn-Schmiedeberg’s Archives of Pharmacology 397(6),* 4025–4034.

Barone, R., Pitruzzella, A., Gammazza, A. M., Rappa, F., Salerno, M., Barone, F., Sangiorgi, C., D'Amico, D., Locorotondo, N., Di Gaudio, F., Cipolloni, L., Di Felice, V., Schiavone, S., Rapisarda, V., Sani, G., Tambo, A., Cappello, F., Turillazzi, E., & Pomara, C. (2017) Nandrolone decanoate interferes with testosterone biosynthesis altering blood–testis barrier components. *Journal of Cellular and Molecular Medicine, 21(8),* 1636–1647.

Behairy, A., El-Sharkawy, N. I., Saber, T. M., Soliman, M. M., Metwally, M. M. M., El-Rahman, C. I. A., Abd-Elhakim, Y. M., & El Deib, M. M. (2020). The modulatory role of vitamin C in boldenone undecylenate induced testicular oxidative damage and androgen receptor dysregulation in adult male rats. *Antioxidants, 9(11),* 1053.

Breuer, M. E., McGinnis, M. Y., Lumia, A. R., & Possidente, B. P. (2001). Aggression in male rats receiving anabolic androgenic steroids: effects of social and environmental provocation. *Hormones and Behavior, 40(3),* 409–418.Grönbladh, A., Johansson, J., Kushnir, M. M., Bergquist, J., & Hallberg, M. (2013). The impact of nandrolone decanoate and growth hormone on biosynthesis of steroids in rats. *Steroids, 78(12-13),* 1192-1199.

Ibrahim, M. M., Sonpol, H. M. A., El Shahat, M. A., Elhawary, A. A., & Bondok, A. A. (2022). Effect of anabolic steroid “Nandrolone” on testes of adult Albino rats: Immunohistochemical and ultrastructural Study. *The Medical Journal of Cairo Univiversity, 90(8),* 2379–2388.

Karbalay-Doust, S., Noorafshan, A., Mesbah Ardekani, F., & Mirkhani, H. (2007). The reversibility of sperm quality after discontinuing nandrolone decanoate in adult male rats. *Asian Journal of Andrology, 9(2),* 235–239.

Kazori, N. (2022). Transgenerational effects of nandrolone decanoate misuse on reproductive system and fertility in male F1 progeny of rat. M.Sc. Thesis, School of Biology, University of Tehran, 80 pages.

Mohamed, H. M., & Mohamed, M. A. H. (2015). Effect of different doses of nandrolone decanoate on lipid peroxidation, DNA fragmentation, sperm abnormality and histopathology of testes of male Wister rats. *Experimental and Toxicologic Pathology, 67(1),* 1–11.

Naraghi, M. A., Abolhasani, F., Kashani, I., Anarkooli, I. J., Hemadi, M., Azami, A., Barbarestani, M., Aitken, R. J., & Shokri, S. (2010). The effects of swimming exercise and supraphysiological doses of nandrolone decanoate on the testis in adult male rats: a transmission electron microscope study. *Folia Morphologica, 69(3),* 138–146.

Oda, S. S., & El-Ashmawy, I. M. (2012). Adverse effects of the anabolic steroid, boldenone undecylenate, on reproductive functions of male rabbits. *International Journal of Experimental Pathology,* 93(3), 172–178.

Saddick, S. Y. (2021). Effect of Nandrolone decanoate induced-oxidative stress on rat testes, prostate, and seminal vesicle: Biochemical, morphometric and histopathological studies. *Saudi Journal of Biological Sciences, 28(1),* 196–203.

Selakovic, D., Joksimovic, J., Jovicic, N., Mitrovic, S., Mihailovic, V., Katanic, J., Milovanovic, D., Pantovic, S., Mijailovic, N., & Rosic, G. (2019). The impact of hippocampal sex hormones receptors in modulation of depressive-like behavior following chronic anabolic androgenic steroids and exercise protocols in rats. *Frontiers in Behavioral Neuroscience, 13,* 19.

Selakovic, D., Joksimovic, J., Zaletel, I., Puskas, N., Matovic, M., & Rosic, G. (2017). The opposite effects of nandrolone decanoate and exercise on anxiety levels in rats may involve alterations in hippocampal parvalbumin–positive interneurons. *PLoS One 12(12),* e0189595.

Shokri, S., Aitken, R. J., Abdolvahhabi, M., Abolhasani, F., Ghasemi, F. M., Kashani, I., Ejtemaeimehr, S., Ahmadian, S., Minaei, B., Naraghi, M. A., & Barbarestani, M. (2010). Exercise and supraphysiological dose of nandrolone decanoate increase apoptosis in spermatogenic cells. Basic & Clinical Pharmacology & Toxicology, *106(4),* 324–330.

Shokri, S., Hemadi, M., Bayat, G., Bahmanzadeh, M., Jafari-Anarkooli, I., & Mashkani, Β. (2014). Combination of running exercise and high dose of anabolic androgenic steroid, nandrolone decanoate, increases protamine deficiency and DNA damage in rat spermatozoa. *Andrologia 46(2),* 184-190.

Zhang, X. W., Zhang, C., Zhang, W., Yang, D., Meng, S., Wang, P., Guo, J., & Liu, D. H. (2016). Suppression of spermatogenesis by testosterone undecanoate-loaded injectable in situ-forming implants in adult male rats. *Asian Journal of Andrology, 18(5),* 791–797.
